# 4-Ethoxy­anilinium perchlorate

**DOI:** 10.1107/S1600536809035041

**Published:** 2009-09-05

**Authors:** Xue-qun Fu

**Affiliations:** a Ordered Matter Science Research Center, Southeast University, Nanjing 210096, People’s Republic of China

## Abstract

In the title compound, C_8_H_12_NO^+^·ClO_4_^−^, there are strong hydrogen bonds between the ammonium groups and the perchlorate O atoms.

## Related literature

This study is a part of systematic investigation of dielectric–ferroelectric materials, including organic ligands (Li *et al.*, 2008 ▶), metal-organic coordination compounds (Hang *et al.*, 2009 ▶) and organic–inorganic hybrids.
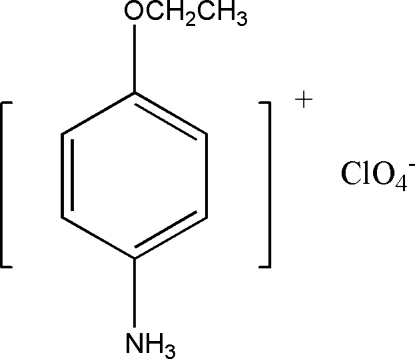


## Experimental

### 

#### Crystal data

C_8_H_12_NO^+^·ClO_4_^−^
*M* *_r_* = 237.64
Monoclinic, 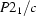

*a* = 5.0663 (10) Å
*b* = 22.601 (5) Å
*c* = 9.2091 (18) Å
β = 91.49 (3)°
*V* = 1054.1 (4) Å^3^
*Z* = 4
Mo *K*α radiation
μ = 0.36 mm^−1^
*T* = 298 K
0.20 × 0.20 × 0.20 mm

#### Data collection

Rigaku SCXmini diffractometer
Absorption correction: multi-scan (*CrystalClear*; Rigaku, 2005 ▶) *T*_min_ = 0.928, *T*_max_ = 0.93
9440 measured reflections
2415 independent reflections
1795 reflections with *I* > 2σ(*I*)
*R*_int_ = 0.055

#### Refinement

*R*[*F* ^2^ > 2σ(*F* ^2^)] = 0.054
*wR*(*F* ^2^) = 0.138
*S* = 1.04
2415 reflections
136 parameters
H-atom parameters constrained
Δρ_max_ = 0.25 e Å^−3^
Δρ_min_ = −0.47 e Å^−3^

### 

Data collection: *CrystalClear* (Rigaku, 2005 ▶); cell refinement: *CrystalClear*; data reduction: *CrystalClear*; program(s) used to solve structure: *SHELXS97* (Sheldrick, 2008 ▶); program(s) used to refine structure: *SHELXL97* (Sheldrick, 2008 ▶); molecular graphics: *SHELXTL* (Sheldrick, 2008 ▶); software used to prepare material for publication: *PRPKAPPA* (Ferguson, 1999 ▶).

## Supplementary Material

Crystal structure: contains datablocks I, global. DOI: 10.1107/S1600536809035041/jh2099sup1.cif

Structure factors: contains datablocks I. DOI: 10.1107/S1600536809035041/jh2099Isup2.hkl

Additional supplementary materials:  crystallographic information; 3D view; checkCIF report

## Figures and Tables

**Table 1:** Hydrogen-bond geometry (Å, °)

*D*—H⋯*A*	*D*—H	H⋯*A*	*D*⋯*A*	*D*—H⋯*A*
N1—H1*A*⋯O4^i^	0.89	2.14	3.019 (3)	167
N1—H1*B*⋯O4^ii^	0.89	2.13	2.981 (3)	161
N1—H1*B*⋯Cl1^ii^	0.89	2.87	3.567 (2)	136
N1—H1*F*⋯O3^iii^	0.89	2.29	2.889 (3)	124
N1—H1*F*⋯O5	0.89	2.29	3.046 (3)	143

Symmetry codes: (i) 
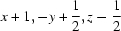
; (ii) 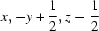; (iii) 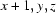.

**Fig. 1.**
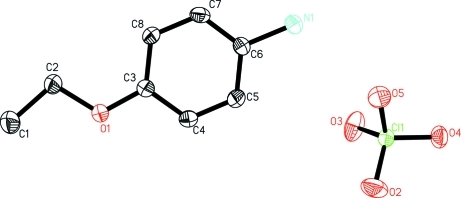
The molecular structure of the title compound, with the atomic numbering scheme. Displacement ellipsoids are drawn at the 30% probability level, and all H atoms have been omitted for clarity.

**Fig. 2.**
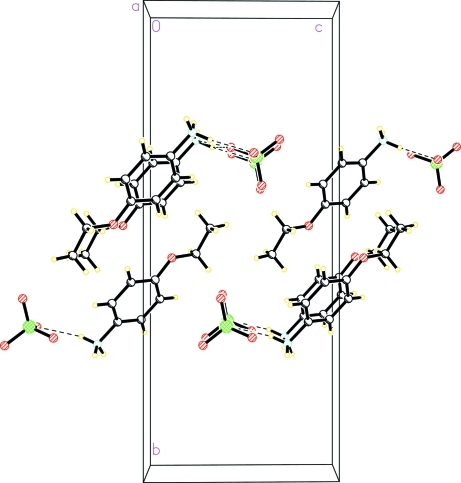
A view of the packing of the title compound, stacking along the a axis. Dashed lines indicate hydrogen bonds.

## supplementary crystallographic information

### Comment

This study is a part of systematic investigation of dielectric-ferroelectric materials, including organic ligands (Li *et al.*, 2008), metal-organic coordination compounds (Hang *et al.*, 2009) and organic-inorganic hybrid. 4-Ethoxyanilinium perchlorate has no dielectric disuniform from 80 K to 450 K, (m.p. 459–460 K).

The asymmetric unit of the title compound is composed of cationic (C_2_H_5_O—C_6_H_4_—NH_3_^+^) and anionic (ClO_4_^-^)(Fig 1). The average Cl—O bond distances and O—Cl—O bond angles are 1.427 (2)Å and 109.46 (14)°, respectively, confirming a tetrahedral configuration. The strong N—H···O hydrogen bonding (Table 1) (N1—H···O3 2.889 (3) Å) make great contribution to the stability of the crystal structure and link the cations and anions to chains along the *a* axis (Fig 2).

### Experimental

Single crystals of 4-ethoxyaniliniumperchlorate are prepared by slow evaporation at room temperature of an ethanol solution of 4-ethoxybenzenamine and perchloric acid.

### Refinement

Positional parameters of all the H atoms were calculated geometrically and were allowed to ride on the C atoms to which they are bonded, with *U*_iso_(H) = 1.2*U*_eq_(C).

### Crystal data

**Table:**

C_8_H_12_NO^+^·ClO_4_^−^	*F*(000) = 496
*M**_r_* = 237.64	*D*_x_ = 1.497 Mg m^−^^3^
Monoclinic, *P*2_1_/*c*	Mo *K*α radiation, λ = 0.71073 Å
Hall symbol: -P 2ybc	Cell parameters from 4042 reflections
*a* = 5.0663 (10) Å	θ = 3.5–27.6°
*b* = 22.601 (5) Å	µ = 0.36 mm^−^^1^
*c* = 9.2091 (18) Å	*T* = 298 K
β = 91.49 (3)°	Prism, colourless
*V* = 1054.1 (4) Å^3^	0.20 × 0.20 × 0.20 mm
*Z* = 4	

### Data collection

**Table:**

Rigaku SCXmini diffractometer	2415 independent reflections
Radiation source: fine-focus sealed tube	1795 reflections with *I* > 2σ(*I*)
graphite	*R*_int_ = 0.055
Detector resolution: 13.6612 pixels mm^-1^	θ_max_ = 27.5°, θ_min_ = 3.5°
ω scans	*h* = −6→6
Absorption correction: multi-scan (*CrystalClear*; Rigaku, 2005)	*k* = −29→29
*T*_min_ = 0.928, *T*_max_ = 0.93	*l* = −11→10
9440 measured reflections	

### Refinement

**Table:**

Refinement on *F*^2^	Primary atom site location: structure-invariant direct methods
Least-squares matrix: full	Secondary atom site location: difference Fourier map
*R*[*F*^2^ > 2σ(*F*^2^)] = 0.054	Hydrogen site location: inferred from neighbouring sites
*wR*(*F*^2^) = 0.138	H-atom parameters constrained
*S* = 1.04	*w* = 1/[σ^2^(*F*_o_^2^) + (0.0663*P*)^2^ + 0.3412*P*] where *P* = (*F*_o_^2^ + 2*F*_c_^2^)/3
2415 reflections	(Δ/σ)_max_ < 0.001
136 parameters	Δρ_max_ = 0.25 e Å^−^^3^
0 restraints	Δρ_min_ = −0.47 e Å^−^^3^

### Special details

**Table:**

Geometry. All e.s.d.'s (except the e.s.d. in the dihedral angle between two l.s. planes) are estimated using the full covariance matrix. The cell e.s.d.'s are taken into account individually in the estimation of e.s.d.'s in distances, angles and torsion angles; correlations between e.s.d.'s in cell parameters are only used when they are defined by crystal symmetry. An approximate (isotropic) treatment of cell e.s.d.'s is used for estimating e.s.d.'s involving l.s. planes.
Refinement. Refinement of *F*^2^ against ALL reflections. The weighted *R*-factor *wR* and goodness of fit *S* are based on *F*^2^, conventional *R*-factors *R* are based on *F*, with *F* set to zero for negative *F*^2^. The threshold expression of *F*^2^ > σ(*F*^2^) is used only for calculating *R*-factors(gt) *etc*. and is not relevant to the choice of reflections for refinement. *R*-factors based on *F*^2^ are statistically about twice as large as those based on *F*, and *R*- factors based on ALL data will be even larger.

### Fractional atomic coordinates and isotropic or equivalent isotropic displacement parameters (Å^2^)

**Table:**

	*x*	*y*	*z*	*U*_iso_*/*U*_eq_	
Cl1	−0.00925 (11)	0.32367 (2)	0.57673 (6)	0.0340 (2)	
N1	0.4143 (4)	0.28041 (9)	0.2574 (2)	0.0385 (5)	
H1A	0.5458	0.2568	0.2319	0.058*	
H1B	0.2657	0.2595	0.2616	0.058*	
H1F	0.4517	0.2961	0.3441	0.058*	
O5	0.2716 (4)	0.31960 (9)	0.5619 (2)	0.0524 (5)	
C7	0.5402 (5)	0.33118 (11)	0.0325 (3)	0.0407 (6)	
H7A	0.6706	0.3028	0.0205	0.049*	
O1	0.2682 (4)	0.46533 (8)	−0.1381 (2)	0.0491 (5)	
C6	0.3806 (5)	0.32820 (10)	0.1488 (3)	0.0343 (5)	
C3	0.3145 (5)	0.41839 (11)	−0.0489 (3)	0.0379 (6)	
O4	−0.0842 (4)	0.28604 (9)	0.6953 (2)	0.0508 (5)	
C8	0.5097 (5)	0.37615 (11)	−0.0678 (3)	0.0412 (6)	
H8A	0.6187	0.3782	−0.1473	0.049*	
O3	−0.1320 (4)	0.30237 (11)	0.4463 (2)	0.0690 (7)	
C4	0.1528 (5)	0.41445 (12)	0.0693 (3)	0.0481 (7)	
H4B	0.0210	0.4425	0.0814	0.058*	
C5	0.1838 (5)	0.36977 (13)	0.1689 (3)	0.0477 (7)	
H5B	0.0749	0.3674	0.2485	0.057*	
C2	0.4325 (7)	0.47354 (13)	−0.2604 (3)	0.0537 (8)	
H2A	0.4204	0.4397	−0.3249	0.064*	
H2B	0.6153	0.4786	−0.2289	0.064*	
O2	−0.0857 (5)	0.38241 (9)	0.6044 (3)	0.0738 (7)	
C1	0.3323 (8)	0.52830 (15)	−0.3361 (4)	0.0750 (11)	
H1C	0.4367	0.5360	−0.4196	0.112*	
H1D	0.3447	0.5613	−0.2707	0.112*	
H1E	0.1513	0.5226	−0.3664	0.112*	

### Atomic displacement parameters (Å^2^)

**Table:**

	*U*^11^	*U*^22^	*U*^33^	*U*^12^	*U*^13^	*U*^23^
Cl1	0.0336 (4)	0.0366 (3)	0.0317 (4)	−0.0012 (2)	0.0009 (2)	0.0012 (2)
N1	0.0387 (13)	0.0395 (12)	0.0367 (12)	−0.0015 (9)	−0.0069 (9)	0.0010 (9)
O5	0.0312 (11)	0.0653 (13)	0.0610 (14)	−0.0010 (8)	0.0053 (9)	0.0067 (10)
C7	0.0417 (15)	0.0362 (14)	0.0441 (15)	0.0104 (11)	0.0001 (12)	−0.0050 (11)
O1	0.0579 (13)	0.0459 (11)	0.0442 (12)	0.0144 (9)	0.0143 (9)	0.0122 (8)
C6	0.0343 (14)	0.0338 (12)	0.0344 (14)	−0.0023 (10)	−0.0052 (10)	0.0007 (10)
C3	0.0384 (15)	0.0376 (13)	0.0379 (15)	0.0027 (10)	0.0016 (11)	0.0014 (10)
O4	0.0505 (13)	0.0586 (12)	0.0437 (11)	−0.0009 (9)	0.0078 (9)	0.0163 (9)
C8	0.0474 (17)	0.0410 (14)	0.0357 (15)	0.0051 (11)	0.0103 (12)	−0.0004 (11)
O3	0.0636 (16)	0.1054 (19)	0.0375 (12)	−0.0328 (13)	−0.0096 (10)	−0.0041 (11)
C4	0.0420 (17)	0.0505 (16)	0.0523 (18)	0.0139 (12)	0.0129 (13)	0.0090 (13)
C5	0.0413 (17)	0.0547 (17)	0.0477 (17)	0.0079 (12)	0.0135 (13)	0.0097 (13)
C2	0.074 (2)	0.0434 (16)	0.0442 (17)	0.0078 (13)	0.0200 (15)	0.0042 (12)
O2	0.0894 (18)	0.0380 (12)	0.0948 (18)	0.0172 (11)	0.0180 (14)	0.0021 (11)
C1	0.113 (3)	0.059 (2)	0.055 (2)	0.0190 (19)	0.026 (2)	0.0138 (16)

### Geometric parameters (Å, °)

**Table:**

Cl1—O2	1.408 (2)	C6—C5	1.386 (4)
Cl1—O3	1.422 (2)	C3—C4	1.382 (4)
Cl1—O5	1.4358 (19)	C3—C8	1.389 (3)
Cl1—O4	1.4426 (19)	C8—H8A	0.9300
N1—C6	1.479 (3)	C4—C5	1.371 (4)
N1—H1A	0.8900	C4—H4B	0.9300
N1—H1B	0.8900	C5—H5B	0.9300
N1—H1F	0.8900	C2—C1	1.502 (4)
C7—C6	1.361 (4)	C2—H2A	0.9700
C7—C8	1.379 (4)	C2—H2B	0.9700
C7—H7A	0.9300	C1—H1C	0.9600
O1—C3	1.358 (3)	C1—H1D	0.9600
O1—C2	1.430 (3)	C1—H1E	0.9600
			
O2—Cl1—O3	110.83 (16)	C7—C8—C3	119.4 (2)
O2—Cl1—O5	110.84 (14)	C7—C8—H8A	120.3
O3—Cl1—O5	108.09 (13)	C3—C8—H8A	120.3
O2—Cl1—O4	109.88 (14)	C5—C4—C3	121.0 (2)
O3—Cl1—O4	108.77 (13)	C5—C4—H4B	119.5
O5—Cl1—O4	108.36 (12)	C3—C4—H4B	119.5
C6—N1—H1A	109.5	C4—C5—C6	118.7 (3)
C6—N1—H1B	109.5	C4—C5—H5B	120.7
H1A—N1—H1B	109.5	C6—C5—H5B	120.7
C6—N1—H1F	109.5	O1—C2—C1	106.0 (2)
H1A—N1—H1F	109.5	O1—C2—H2A	110.5
H1B—N1—H1F	109.5	C1—C2—H2A	110.5
C6—C7—C8	120.3 (2)	O1—C2—H2B	110.5
C6—C7—H7A	119.8	C1—C2—H2B	110.5
C8—C7—H7A	119.8	H2A—C2—H2B	108.7
C3—O1—C2	118.9 (2)	C2—C1—H1C	109.5
C7—C6—C5	121.1 (2)	C2—C1—H1D	109.5
C7—C6—N1	120.5 (2)	H1C—C1—H1D	109.5
C5—C6—N1	118.4 (2)	C2—C1—H1E	109.5
O1—C3—C4	115.5 (2)	H1C—C1—H1E	109.5
O1—C3—C8	125.0 (2)	H1D—C1—H1E	109.5
C4—C3—C8	119.5 (2)		
			
C8—C7—C6—C5	0.4 (4)	O1—C3—C4—C5	−178.6 (2)
C8—C7—C6—N1	−179.4 (2)	C8—C3—C4—C5	0.6 (4)
C2—O1—C3—C4	178.1 (3)	C3—C4—C5—C6	−0.2 (4)
C2—O1—C3—C8	−1.1 (4)	C7—C6—C5—C4	−0.3 (4)
C6—C7—C8—C3	0.0 (4)	N1—C6—C5—C4	179.5 (2)
O1—C3—C8—C7	178.6 (2)	C3—O1—C2—C1	179.9 (3)
C4—C3—C8—C7	−0.5 (4)		

### Hydrogen-bond geometry (Å, °)

**Table:**

*D*—H···*A*	*D*—H	H···*A*	*D*···*A*	*D*—H···*A*
N1—H1A···O4^i^	0.89	2.14	3.019 (3)	167
N1—H1B···O4^ii^	0.89	2.13	2.981 (3)	161
N1—H1B···Cl1^ii^	0.89	2.87	3.567 (2)	136
N1—H1F···O3^iii^	0.89	2.29	2.889 (3)	124
N1—H1F···O5	0.89	2.29	3.046 (3)	143

Symmetry codes: (i) *x*+1, −*y*+1/2, *z*−1/2; (ii) *x*, −*y*+1/2, *z*−1/2; (iii) *x*+1, *y*, *z*.
